# Case of Compound Fracture of the Os Humeri

**Published:** 1814-06

**Authors:** John Wayte

**Affiliations:** Calne


*For the Medical and Physical Journal.*

### *Case of Compound Fracture of the Os Humeri; by* Mr. WAYTE.

IN the evening of September 15th, 1813, Thomas Torrence, fifteen years of age, was thrown between the leading horses of a loaded broad-wheel waggon: every horse walked over him without inflicting the least injury; but the wheels coming obliquely over his os humeri, produced a most terrible compound fracture. After being conveyed home, and his clothes removed, there were exhibited appearances precisely as follow:—A large lacerated wound internally, and at the more superior part of the biceps-flexor muscle, where also was found the brachial artery totally obliterated. The fractured bone was unfavourably near the condyls, and evidently in a shattered state; but neither over nor under it did there appear any present marks of severe contusion, which shortly after presented a most malignant picture. The hæmorrhage was trifling; no pulse, of course, was to be felt at the the wrist; and, in fact, the limb apparently lifeless. All due consideration was given as to saving or amputating such a limb. On the one hand, for amputation was adduced the following reasons: That mortification was greatly to be expected from the obliteration of an artery where large anastomoses could not be calculated on; and again, that the arm might probably be useless, provided life could be sustained. On the other hand, for saving it were considered his youth, good habit of body, probable anastomoses and enlargement for sufficient support; Mr. A. Cooper's rule in mortification, that if it arises from injury amputation may be performed at any time, and also when apparent that nature would have sunk under the discharge. The arm being placed in an easy and proper position, a decision was delayed till the morrow, when the arm to his fingers' ends being found to possess a natural heat, colour, elasticity, and sensation, it was resolved to attempt a preservation.

From having often witnessed the bad effects of hot linseed or other poultices, and hot fomentations, to fractures, conceiving that they excited by far too much local inflammation and discharge, with proportionate constitutional irritation, I pursued, as has ever been my plan, a most simple antiphlogistic treatment. Two sutures were used at the wound; dry lint, a soap cerate plaster, tailed bandage and splints, with saturnine lotion sparingly, were the first applications. I would not deplete by venæsection, considering that nature had much to effect, and would require a long time in effecting. His bowels were kept soluble, and an opiate given each night to the 21st, during which period no symptoms of high irritation presented themselves, having alternate ease and pain, with a pulse beating evenly soft from 80 to 96.

On the 23d there were indications of very extensive sloughing, without a power of ascertaining the depth; but in other respects he was tolerably easy, and calmly cheerful.

On the 25th I found the sloughs inclined to separate, and their superficial extent was surprising. Underneath it reached from the inner condyle almost up to the axilla, in breadth exceeding two inches. On and from the outer condyle it extended half way up the humerus, and equal to that in breadth, literally leaving scarcely ought but wound.

By the 28th the under slough had come away, proving to be only skin-deep; but as the discharge was becoming copious, fœtid, and sanious, with considerable languor, the bark and cordial confection were freely given.

On the 29th feels comfortable, pulse good, discharge purulent from beneath, still sanious above; am under the necessity of removing all pressure from the internal condyle, as as the wound extends over it, and pressure causes excruciating pain.

The sloughing process having proceeded well, there was brought away on the 3d of October a large muscular portion, seemingly from the triceps extensor and origin of the supinator radii longus.

On the 4th a second large portion with some fragments separated, being part of the biceps flexor, which exposed a cutting edge of bone of the superior portion, and granulations were seen plentifully arising.

By the 8th every dead portion was come away, a clean healthy surface left, and in places a disposition to cicatrize.

10th.—One small piece of bone (the above-mentioned exposed edge) has exfoliated; he is going on well, takes muriatic acid in bark, eats, drinks, and sleeps.

On 16th was permitted to come down stairs, with proper assistance and precautions, although the bone had not united; but I considered myself warranted in pursuing such a measure, from knowing that my splints were so constructed as to preserve perfect approximation and quietude, and of the salutary effects of change of air and position no one will doubt.

From that period has he been in a progressive state of amendment, with the wounds all but healed by the end of February, and capable of executing many things with his arm; yet from commencing hard work too soon (such as wheeling and carrying for a gardener), an ulcerative process supervened and occasioned some delay, the effects of which, by refraining from such duty, are overcome, and in all probability no wound will be seen at the end of this month. It must be observed that opiates and bark were continued a long time; and that the dressings, according to circumstances, were combinations of cerate of resin and calamine, of cerate of calamine and ointment of nitrated silver.

*Calne, April* 14, 1814. JOHN WAYTE.

